# Treating Parry-Romberg syndrome with hyaluronic acid: insights after 2.5 years of successive treatments^☆^

**DOI:** 10.1016/j.abd.2024.08.007

**Published:** 2025-03-13

**Authors:** Roberta Vasconcelos-Berg, Barbara Varella Maire, Alexander A. Navarini

**Affiliations:** a Margarethenklinik, University Hospital of Basel, Basel, BS, Switzerland; b Department of Dermatology, University Hospital of Basel, Basel, BS, Switzerland

*Dear Editor,*

An 18-year-old female patient with a previous diagnosis of Parry-Romberg syndrome (PRS) since childhood was referred to our clinic for facial symmetrization. The patient was on methotrexate 15 mg/week, with no evidence of disease progression in recent years. She had previously undergone autologous fat grafting with ephemeral and unsatisfactory results.

On dermatological examination, she presented signs of cutaneous and muscular atrophy on the right side of her face, resulting in asymmetry in the height of the eyebrows, and atrophy in the right zygomatic, subocular, mandibular, and mental regions. In the right subocular region, cutaneous atrophy manifested as hypopigmentation and exposure of the dermal vascular network (Fig. 1A).

**Figure 1:**
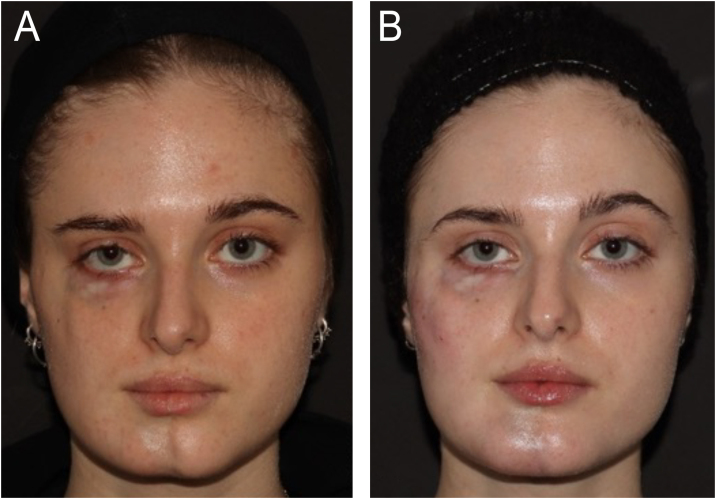
(A) Baseline in April 2021 showing skin and deep planes atrophy of the right hemiface in a patient with Parry-Romberg syndrome. (B) The patient after the last filler treatment in February 2024, showed improved facial symmetry, especially in the zygomatic region, tear trough, and chin. Between the first and last photos, 12 treatments were performed with a median volume of 2 mL per session, every 60 days. The tear trough region was treated with a 25 G blunt cannula using Restylane Lidocaine deeply, and the zygomatic region, and chin were treated both supraperiosteally (with a 27 G needle using Restylane Lyft) and in the deep subcutaneous region (with blunt cannula 22 G using Restylane Defyne).

For facial symmetrization, hyaluronic acid filler (Restylane, Galderma Laboratoires, Upsala) was chosen.

From the first treatment in April 2021, where 6.6 mL of Hyaluronic Acid (HA) was injected, to February 2024, the patient returned for 12 additional sessions. The time between treatments ranged from 46 to 153 days (median 60 days), and the volume of hyaluronic acid per session varied between 0.75 and 3 mL (median 2 mL). All treatments were guided by ultrasound.

The hyaluronic acid injections were administered only to the affected hemiface. The treated regions included the lateral and medial zygomatic areas, tear trough, and chin. The treatment details are described in Fig. 1B.

In two sessions, hyaluronidase was needed in the chin area to correct irregularities caused by hyaluronic acid migration.

During one treatment in the chin area, signs of local ischemia were observed (Fig. 2). However, the patient reported that this same region periodically and spontaneously exhibited these signs even before the first hyaluronic acid injection. Indeed, Doppler ultrasound performed during the procedure did not show arterial occlusion. The symptoms regressed after a few hours of treatment, even without hyaluronidase injection.

**Figure 2:**
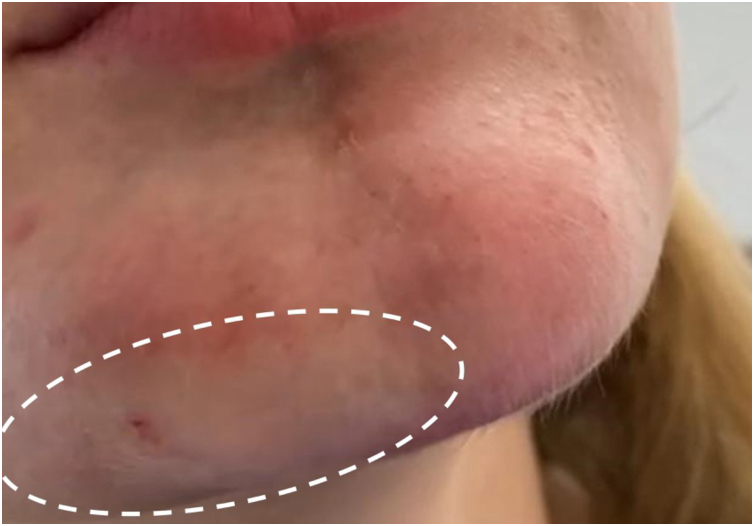
Blanching of the right lateral chin region (dotted area) during hyaluronic acid injection, indicating likely ischemia in the area.

Parry-Romberg Syndrome is a rare acquired neurocutaneous disease of unknown etiopathogenesis, typically characterized by progressive hemifacial atrophy. It is commonly characterized as an autoimmune disorder within the spectrum of diseases associated with localized scleroderma *en coup de sabre*. This classification is substantiated by evidence of inflammatory histopathology, the presence of serum autoantibodies, the coexistence of other autoimmune conditions, and positive responses to immunosuppression. Extracutaneous disease manifestations, including neurologic, ocular, and oral pathology, are common,^1^ but was not the case with our patient.

The classic treatment for facial symmetrization is autologous fat grafting, which was not indicated in our case due to the patient's previous dissatisfaction. Hyaluronic acid filling has been previously reported in the literature in four cases of PRS.^2^, ^3^, ^4^ In none of them the treatment was guided by ultrasound, and none reported to be on immunosuppressive treatment. There was no report of disease progression associated with aesthetic treatment. No infections, rejections, or other complications related to this treatment were observed.

The use of hyaluronic acid in patients with morphea/scleroderma spectrum diseases was reviewed in 2020.^5^ The retrospective analysis of 488 reported cases, treated with different brands and types of HA, did not result in disease progression.

Some peculiarities were observed in the treatment of this patient: The average time for complete absorption of hyaluronic acid after injection varies depending on the molecular composition of HA,^6^ but treatment repetition is expected to be between 6- and 12 months for healthy individuals. In our case, treatment was repeated approximately every two months. Fig. 3 shows the volume reduction in the right zygomatic region only 80 days after the hyaluronic acid injection. This leads us to question whether there is a higher turnover of injected HA as part of the disease in PRS.

**Figure 3:**
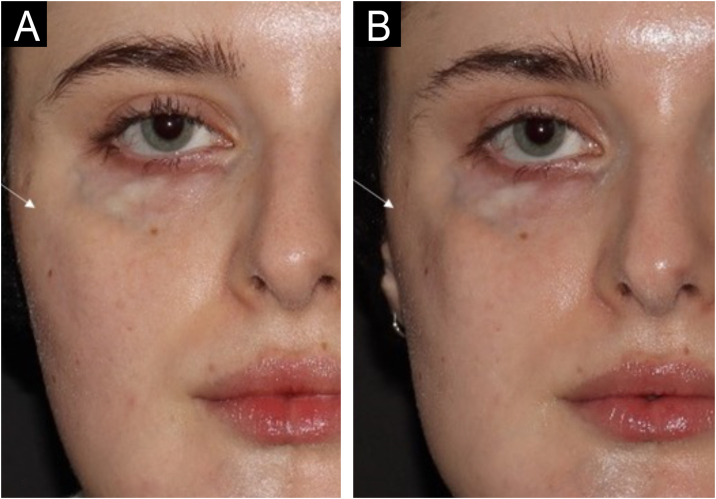
The same patient with an 80-day interval. On the day of the first photo (A), 0.9 mL of Restylane Defyne was injected into the right zygomatic region (result not shown). Despite this, in photo B, 80 days after the injection, the zygomatic region (white arrow) appears visibly more depressed than in the previous photo. This absorption pattern was observed over the three years of follow-up, which may represent accelerated hyaluronic acid absorption.

Besides the difficulty of moving the cannula during the procedure, tissue rigidity, associated with periodic vasospasms, could pose a higher risk of vascular compression by HA. Additionally, the spontaneous cutaneous vasospasms in these patients may be a confounding factor for real vascular occlusion by HA during the procedure. Performing the procedure guided by ultrasound can help in differentiation. Tissue rigidity could also explain the frequent migration of HA, which, when compressed by muscle movement against adjacent tissues, might herniate to areas with less resistance. This difficulty may be even greater in the chin area, where muscle fibers and subcutaneous fatty tissue are naturally interwoven, making the tissue more compact. In this sense, favoring products with greater tissue integration could be a good treatment strategy.

Considering the importance of facial symmetrization in the quality of life and given the low incidence of adverse effects, hyaluronic acid can be a good option in PRS. However, due to the technical difficulty associated with treating PRS, it is recommended to be performed by experienced professionals with extensive anatomical knowledge and prepared to handle vascular and infectious events.

## Financial support

None declared.

## Authors’ contributions

Roberta Vasconcelos-Berg: The study concept and design; writing of the manuscript and critical review of important intellectual content; final approval of the final version of the manuscript.

Barbara Varella Maire: Data collection, analysis and interpretation of data; critical review of the literature.

Alexander A. Navarini: Writing of the manuscript and critical review of important intellectual content; final approval of the final version of the manuscript.

## Conflicts of interest

Roberta Vasconcelos-Berg is a speaker and consultant for Galderma Laboratoires. Barbara Varella Maire and Alexander A. Navarini have no conflict of interest to declare.
